# Supplementary material for: Relation of prior statin and anti-hypertensive use to severity of disease among patients hospitalized with COVID-19: Findings from the American Heart Association’s COVID-19 Cardiovascular Disease Registry
Source: PLoS One. 2021 Jul 15;16(7):e0254635. doi: 10.1371/journal.pone.0254635 (PMC8281996; doi:10.1371/journal.pone.0254635)
Supplement: S1 Appendix — (PDF) [file pone.0254635.s001.pdf]

S1 Appendix

Competing Risks Sensitivity Analysis for Time to Severe Outcome

As a sensitivity analysis a competing-risks analysis was used to investigate the association of the exposure of interest (use of statin or anti-HTN) with time to onset of severe disease or to recovery, whichever came first; the starting timepoint was the symptom onset date for COVID-19. For each of these 2 outcomes, a Cox proportional hazards regression model was used in which the competing outcome was treated as a censoring event. We adjusted for the same potential confounders as in the mixed effect logistic regression analyses. The proportional hazards assumption was tested by examining Schoenfeld residuals.

A total of 8,929 patients had complete data on date of COVID-19 symptom onset, date of admission/discharge, and date of ICU admission, and were included in time-to-event analyses. Multivariable Cox proportional hazards regression was used to investigate the association of each of the two competing outcomes (either severe disease or recovery) with use of statin and/or anti-HTN, treating the competing outcome as a censoring event and adjusting for potential confounders. Compared to taking neither statin nor anti-HTN, patients taking both classes of medication had a decreased rate of development of severe disease (cause-specific adjusted hazard ratio [aHR] for severe disease 0.84, 95% CI 0.75-0.94); taking statin alone (aHR for severe disease 0.88, 95% CI 0.76-1.02) or anti-HTN alone (aHR for severe disease 0.97, 95% CI 0.86-1.08) trended toward reduced rate of severe disease, but were not statistically significant. Other covariates related to increased rate of severe COVID-19 were male sex, older age, obesity, CKD, pulmonary disease, diabetes, CVD and hypertension. However, the proportional hazards assumption was violated for multiple variables; further modeling is required.
