# Supplementary material for: Relation of prior statin and anti-hypertensive use to severity of disease among patients hospitalized with COVID-19: Findings from the American Heart Association’s COVID-19 Cardiovascular Disease Registry
Source: PLoS One. 2021 Jul 15;16(7):e0254635. doi: 10.1371/journal.pone.0254635 (PMC8281996; doi:10.1371/journal.pone.0254635)
Supplement: S1 Table — (PDF) [file pone.0254635.s002.pdf]

S1 Table. Characteristics of patients who survived compared with those with in-hospital death/discharge to hospice

	Alive (n=8329)	Death (n=2212)	OR	95% CI	p
**Male**	4,553 (55%)	1,262 (57%)	1.10	1.00,1.21	0.05
**Age (years)***	63.8 ± 13.5	75.1 ± 12.9	1.85	1.78,1.92	<0.001
**Insurance status**					
Medicare (over age 65)	3,808 (46%)	1,754 (79%)	6.91	5.76,8.36	<0.001
Public	1,790 (21%)	274 (12%)	2.30	1.85,2.87	<0.001
No insurance/unknown	689 (8%)	48 (2%)	1.05	0.73,1.48	0.79
Private	2,042 (25%)	136 (6%)	REF	REF	REF
**Race**					
Hispanic	2,272 (27%)	502 (23%)	0.69	0.61,0.78	<0.001
Black	2,234 (27%)	524 (24%)	0.73	0.65,0.83	<0.001
Other	979 (12%)	273 (12%)	0.87	0.74,1.01	0.08
Non-Hispanic White	2,844 (34%)	913 (41%)	REF	REF	REF
**Medication history†**					
Statin	888 (11%)	369 (17%)	1.24	1.13,1.37	<0.001
ACE inhibitor	1,461 (18%)	370 (17%)	0.94	0.83,1.07	0.38
ARB	1,150 (14%)	326 (15%)	1.08	0.94,1.23	0.27
Beta blocker	2,213 (27%)	769 (35%)	1.47	1.33,1.63	<0.001
Calcium channel blocker	1,889 (23%)	620 (28%)	1.33	1.19,1.48	<0.001
Diuretic	1,483 (18%)	492 (22%)	1.32	1.17,1.48	<0.001
Other antihypertensive	509 (6%)	172 (8%)	1.30	1.08,1.55	0.006
Antiplatelet agent	2,290 (27%)	820 (37%)	1.55	1.41,1.72	<0.001
Anticoagulant	888 (11%)	369 (17%)	1.68	1.47,1.92	<0.001
**Comorbidities†**					
Cardiovascular disease	2,339 (28%)	1,021 (46%)	2.20	1.99,2.42	<0.001
Hypertension	5,283 (63%)	1,693 (77%)	1.88	1.69,2.10	<0.001
Diabetes	3,254 (39%)	973 (44%)	1.22	1.11,1.35	<0.001
Cancer	936 (11%)	339 (15%)	1.43	1.25,1.64	<0.001
Chronic kidney disease	1,096 (13%)	500 (23%)	1.93	1.71,2.17	<0.001
Dyslipidemia	3,193 (38%)	1,010 (46%)	1.35	1.23,1.49	<0.001
Obesity (BMI >30 kg/m2)	3,613 (43%)	719 (33%)	0.63	0.57,0.69	<0.001
Smoking or vaping	555 (7%)	166 (8%)	1.14	0.94,1.36	0.1694
Immune disorder	397 (5%)	107 (5%)	1.02	0.81,1.27	0.9107
Pulmonary disease	1,474 (18%)	453 (20%)	1.20	1.06,1.35	0.003
Other comorbidities	22 (0%)	4 (0%)	0.68	0.17,2.02	0.6323
**Outcomes**					
Intensive care unit	1,916 (23%)	1,238 (56%)	4.25	3.85,4.70	<0.001
Mechanical ventilation	944 (11%)	1,098 (50%)	7.71	6.91,8.60	<0.001

*Change in odds per 10-year increment in age

†OR for death/discharge to hospice compared to absence of the indicated medication or comorbidity

ACE = angiotensin-converting enzyme; ARB = angiotensinogen II receptor blocker; BMI = body mass index; CI = confidence interval; OR = odds ratio for death.
