# Supplementary material for: Relation of prior statin and anti-hypertensive use to severity of disease among patients hospitalized with COVID-19: Findings from the American Heart Association’s COVID-19 Cardiovascular Disease Registry
Source: PLoS One. 2021 Jul 15;16(7):e0254635. doi: 10.1371/journal.pone.0254635 (PMC8281996; doi:10.1371/journal.pone.0254635)
Supplement: S2 Table — (PDF) [file pone.0254635.s003.pdf]

S2 Table. Characteristics of patients with severe versus non-severe COVID-19

	Non-Severe (n=6380)	Severe (n=4161)	OR	95% CI	p
**Male**	3,392 (53%)	2,423 (58%)	1.23	1.13,1.33	<0.001
**Age (years)***	64.1 ± 13.9	69.4 ± 13.9	1.32	1.28,1.35	<0.001
**Insurance status**					
Medicare (over age 65)	2,949 (46%)	2,613 (63%)	2.04	1.83,2.27	<0.001
Public	1,363 (21%)	701 (17%)	1.18	1.04,1.35	0.01
No insurance/unknown	550 (9%)	187 (4%)	0.78	0.64,0.95	0.01
Private	1,518 (24%)	660 (16%)	REF	REF	REF
**Race**					
Hispanic	1,826 (29%)	948 (23%)	0.64	0.58,0.71	<0.001
Black	1,713 (27%)	1,045 (25%)	0.75	0.68,0.83	<0.001
Other	770 (12%)	482 (12%)	0.77	0.67,0.88	<0.001
Non-Hispanic White	2,071 (32%)	1,686 (41%)	REF	REF	REF
**Medication history†**					
Statin	2,609 (41%)	1,840 (44%)	1.15	1.06,1.24	<0.001
ACE inhibitor	1,098 (17%)	733 (18%)	1.03	0.93,1.14	0.60
ARB	880 (14%)	596 (14%)	1.04	0.93,1.17	0.45
Beta blocker	1,683 (26%)	1,299 (31%)	1.27	1.16,1.38	<0.001
Calcium channel blocker	1,390 (22%)	1,119 (27%)	1.32	1.2,1.45	<0.001
Diuretic	1,130 (18%)	845 (20%)	1.18	1.07,1.31	<0.001
Other antihypertensive	392 (6%)	289 (7%)	1.14	0.97,1.34	0.11
Antiplatelet agent	1,745 (27%)	1,365 (33%)	1.30	1.19,1.41	<0.001
Anticoagulant	678 (11%)	579 (14%)	1.36	1.21,1.53	<0.001
**Comorbidities†**					
Cardiovascular disease	1,775 (28%)	1,585 (38%)	1.60	1.47,1.74	<0.001
Hypertension	3,983 (62%)	2,993 (72%)	1.54	1.42,1.68	<0.001
Diabetes	2,446 (38%)	1,781 (43%)	1.20	1.11,1.3	<0.001
Cancer	734 (12%)	541 (13%)	1.15	1.02,1.3	0.02
Chronic kidney disease	829 (13%)	767 (18%)	1.51	1.36,1.69	<0.001
Dyslipidemia	2,409 (38%)	1,794 (43%)	1.25	1.15,1.35	<0.001
Obesity (BMI >30 kg/m2)	2,675 (42%)	1,657 (40%)	0.92	0.85,0.99	0.03
Smoking or vaping	424 (7%)	297 (7%)	1.08	0.92,1.26	0.34
Immune disorder	310 (5%)	194 (5%)	0.96	0.79,1.15	0.67
Pulmonary disease	1,077 (17%)	850 (20%)	1.26	1.14,1.4	<0.001
Other comorbidities	15 (0%)	11 (0%)	1.12	0.47,2.62	0.84
**Outcomes**					
Intensive care unit	0 (0%)	3,154 (76%)			
Mechanical ventilation	0 (0%)	2,042 (49%)			
Death or hospice	0 (0%)	2,212 (53%)			

*Change in odds per 10-year increment in age

†OR for severe outcome (death/discharge to hospice, mechanical ventilaion, intensive care unit) compared to absence of the indicated medication or comorbidity

Severe disease includes need for intensive care unit or mechanical ventilation, or death.
ACE = angiotensin-converting enzyme; ARB = angiotensinogen II receptor blocker, BMI = body mass index;
CI = confidence interval; OR = odds ratio for severe disease.
