# Supplementary figures and images for: Relation of prior statin and anti-hypertensive use to severity of disease among patients hospitalized with COVID-19: Findings from the American Heart Association’s COVID-19 Cardiovascular Disease Registry
Source: PLoS One. 2021 Jul 15;16(7):e0254635. doi: 10.1371/journal.pone.0254635 (PMC8281996; doi:10.1371/journal.pone.0254635)

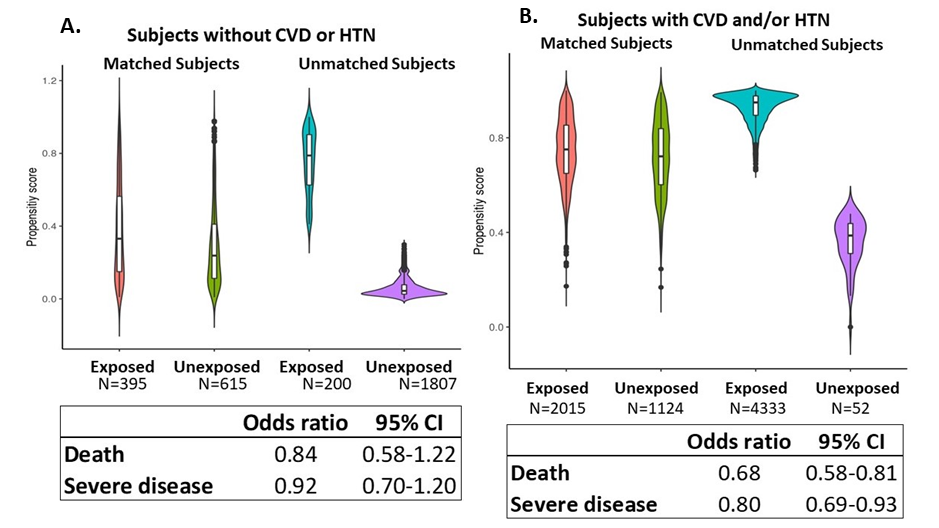

Supplement: S1 Fig — Plot of propensity scores by matching status for (A) “healthy” patients, with neither a history of cardiovascular disease nor hypertension, and (B) patients with a history of cardiovascular disease and/or hypertension. Exposed versus unexposed refers to history of statin and/or anti-hypertensive medication use prior to hospitalization. The y axis shows the estimated probability of medication use (i.e. the propensity score), from the propensity score logistic regression model. Tables show the odds ratio (OR) for risk of death (in-hospital death or discharge to hospice) or severe disease (need for intensive care unit or mechanical ventilation, or death), comparing exposed to matched unexposed patients. (TIF) [file pone.0254635.s004.tif]
